# The impact of donor diabetes on recipient postoperative complications, renal function, and survival rate in deceased donor kidney transplantation: a single-center analysis

**DOI:** 10.1080/0886022X.2024.2391067

**Published:** 2024-08-23

**Authors:** Qi Chen, Jiayu Guo, Shangting Han, Tianyu Wang, Kang Xia, Bo Yu, Yiting Liu, Tao Qiu, Jiangqiao Zhou

**Affiliations:** a Department of Organ Transplantation, Renmin Hospital of Wuhan University, Wuhan University, Wuhan, Hubei, China; b Department of Urology, Renmin Hospital of Wuhan University, Wuhan University, Wuhan, Hubei, China

**Keywords:** Deceased donor kidney transplantation, diabetic donor, renal pathology score, kidney function, delayed graft function

## Abstract

As the global incidence of diabetes rises and diagnoses among younger patients increase, transplant centers worldwide are encountering more organ donors with diabetes. This study examined 80 donors and 160 recipients, including 30 donors with diabetes (DD) and their 60 recipients (DDR). The control group comprised 50 non-diabetic donors (ND) and 100 recipients (NDR). We analyzed clinical, biochemical, and pathological data for both diabetic and control groups, using logistic regression to identify risk factors for delayed graft function (DGF) after kidney transplantation. Results showed that pre-procurement blood urea nitrogen levels were significantly higher in DD [18.20 ± 10.63 *vs.* 10.86 ± 6.92, *p* = 0.002] compared to ND. Renal pathological damage in DD was notably more severe, likely contributing to the higher DGF incidence in DDR compared to NDR. Although DDR had poorer renal function during the first three months post-transplant, both groups showed similar renal function thereafter. No significant differences were observed in 1-year or 3-year mortality rates or graft failure rates between DDR and NDR. Notably, according to the Renal Pathology Society (RPS) grading system, kidneys from diabetic donors with a grade > IIb are associated with significantly lower postoperative survival rates. Recipient gender [OR: 5.452 (1.330–22.353), *p* = 0.013] and pre-transplant PRA positivity [OR: 34.879 (7.698–158.030), *p* < 0.001] were identified as independent predictors of DGF in DDR. In conclusion, transplant centers may consider utilizing kidneys from diabetic donors, provided they are evaluated pathologically, without adversely impacting recipient survival and graft failure rates.

## Introduction

The prevalence of end-stage renal disease (ESRD) is increasing globally. Compared to dialysis, kidney transplantation offers patients extended survival and an improved quality of life, while also reducing the long-term financial burden on patients [1,2]. Although the majority of countries have been actively promoting Donation after Circulatory Death (DCD), the number of patients on the kidney transplant waiting list continues to rise annually, with many individuals succumbing while awaiting a transplant [3,4]. Given the growing shortage of ideal donors, the Crystal City Conference in Virginia in 2001 advocated for broadening the sources of donated kidneys and encouraged the utilization of marginal donor kidneys [5]. Naturally, kidneys from donors who are advanced in age, have a history of hypertension, infectious diseases, or diabetes are increasingly being considered [6]. Diabetes was once regarded as a relative contraindication for kidney donation. Sung et al. utilized the Scientific Registry of Transplant Recipients (SRTR)/Organ Procurement and Transplantation Network (OPTN) database to reveal that, between 1999 and 2005, the rate of kidney abandonment among donors with a history of diabetes was 1.89 times higher than that of donors without such a history (*p* < 0.0001) [7].

Diabetic nephropathy is characterized by the accumulation of extracellular matrix in the glomeruli and tubulointerstitium, thickening of renal vessels, and glomerular vitrifications [8]. Under hyperglycemic conditions, renal cells demonstrate altered glucose uptake and metabolism, resulting in the excessive formation of advanced glycation end-products (AGEs) and reactive oxygen species (ROS). Consequently, this triggers the activation of several downstream signaling pathways, including protein kinase C (PKC), transforming growth factor-beta-mitogen-activated protein kinase (TGF-β-MAPK), and Janus kinase-signal transducer and activator of transcription (JAK-STAT) pathways [9–12]. Simultaneously, hyperglycemia can induce renal vascular endothelial dysfunction by decreasing nitric oxide (NO) release from endothelial cells and exacerbating oxidative stress. This leads to the overproduction of inflammatory factors, ultimately impairing endothelial repair and disrupting normal angiogenesis [13]. Together, these mechanisms contribute to the development of glomerulosclerosis, tubular atrophy, renal interstitial fibrosis, and arteriolar stenosis in patients with diabetes.

However, there currently appears to be no research demonstrating a significant impact of using kidneys from diabetic patients on recipient survival rates. In fact, not only is the prevalence of diabetes high in developed countries, but it is also markedly increasing in developing nations [14–16]. This suggests that diabetic donors are likely to become more common in the future. This study conducted a retrospective analysis of data from deceased donor kidney transplants (DDKT) at Wuhan University People’s Hospital between 1 May 2018 and 30 April 2021. The objective was to evaluate the effects of diabetic donor kidneys on recipient postoperative complications, renal function, and both short-term and long-term outcomes.

## Methods

### Study participants

DDKT donors and recipients were included in this study during the period from 1 May 2018 to 30 April 2021 in Renmin Hospital of Wuhan University. The donation criteria for donors are (1) clear identity of the donor; (2) age not exceeding 65 years old; (3) inactive human immunodeficiency virus (human immunodeficiency virus, HIV) infection; (4) no history of drug abuse; (5) no malignant melanoma, metastatic malignant tumor, or incurable malignant tumor; and (6) no active, untreated systemic bacterial, viral or fungal infection. (7) The function of donated organs is basically normal. (8) Severe and irreversible cardiopulmonary or nerve injuries have reached the diagnostic criteria of brain death or are expected to die in 60 min after the withdrawal of life support therapy. Recipients include first-time or previous recipients of kidney transplants.

### Important variables, diagnostic criteria, and clinical outcomes

All donor and recipient clinical data were managed by two dedicated follow-up physicians, including information from donor admission until acquisition and preoperative and postoperative follow-up of recipients.

The diagnostic criteria of donor diabetes are (1) a clear history of diabetes before donation; or (2) glycosylated hemoglobin (HbA1c) > 6.5% [17,18].

DGF is defined as the need for dialysis within 1 week after renal transplantation [19]. Renal allograft failure is defined as postoperative renal allograft dysfunction, and the recipient needs renal replacement therapy and/or retransplantation [20].

PRA > 10% means that the recipient’s group reactive antibodies class I or class II before transplantation are >10%. Kerman et al. first reported that the incidence of acute rejection after renal transplantation in renal transplant recipients with preoperative PRA > 10% could reach 70% [21]. Therefore, most transplant centers in China regard PRA > 10% as positive, which is closely related to the occurrence of rejection after renal transplantation.

The donor kidneys were routinely taken for pathological examination, and the glomeruli, renal tubules, renal vessels, and renal interstitium were scored. No pathological changes were observed as 0. When more than 50% of glomerulosclerosis, the glomerular score is 3; when more than 50% of renal tubules atrophy, the renal tubule score is 3; when more than 50% of the renal parenchyma is replaced by connective tissue, the renal interstitial score is 3; when the wall thickness exceeds the lumen diameter or lumen occlusion, the renal vascular score is 3. Finally, the sum of the four scores, that is, the total score of Remuzzi, was used to evaluate the quality of donor kidney. We specify that the total Remuzzi score of donor kidney is 0–3 as a low score, and higher than 4 as a high score [22].

The Renal Pathology Society established the classification of diabetic nephropathy in 2010. 0: no diabetic changes under light or electron microscope; I: there was no obvious change under light microscope, but glomerular basement membrane thickened under electron microscope; IIa: mild Mesangial dilatation under light microscope; IIb, obvious Mesangial dilatation under light microscope; III, nodular sclerosis (Kimmelstiel–Wilson lesions): at least one glomerulus with nodular increase in mesangial matrix (Kimmelstiel–Wilson) without changes described in IV; IV, advanced diabetic glomerulosclerosis: more than 50% global glomerulosclerosis with other clinical or pathologic evidence that sclerosis is attributable to diabetic nephropathy [23].

The primary clinical outcomes of this study were recipient mortality and graft failure following kidney transplantation. The secondary outcome was the incidence of DGF in recipients post-surgery.

### Data collection

The collection of clinical data includes pre-donation data of donors and clinical data of recipients. The donor data included: (1) clinical data, such as sex, age, body mass index, and blood type, (2) complications and primary disease, (3) relevant laboratory tests, such as initial serum creatinine, initial urea, initial eGFR, initial albumin, terminal serum creatinine, terminal urea, terminal eGFR, terminal albumin, terminal hemoglobin, terminal urinary protein and (4) Kidney pathology information, such as Remuzzi score and RPS grade. The recipient data included: (1) sex, age, body mass index, blood type, and other clinical data; (2) transplantation data, including transplant times, PRA, HLA mismatch times; (4) complications and primary disease; (5) postoperative laboratory examination indexes, such as hemoglobin, ALT, AST, 24 h serum creatinine and eGFR, 3 days serum creatinine and eGFR, 7 days serum creatinine and eGFR, 14 days serum creatinine and eGFR, 1 month serum creatinine and eGFR, 3 months serum creatinine and eGFR, 6 months serum creatinine and eGFR, 1 year serum creatinine and eGFR, 2 years serum creatinine and EGFR, 3 years serum creatinine and eGFR, 4 years serum creatinine and eGFR, 5 years serum creatinine and eGFR. (6) Postoperative complications, including DGF and renal allograft failure. (7) Three-year postoperative mortality.

### Immunosuppressive therapy

Our center employs a triple immunosuppressive regimen for kidney transplant recipients, comprising tacrolimus, mycophenolate mofetil, and glucocorticoids. The initial dosage of tacrolimus is 0.05–0.15 mg/kg/day, with a 12-h trough concentration target of 8–12 ng/ml during the first month, 6–10 ng/ml from 1 to 3 months, 5–10 ng/ml from 4 to 12 months, and 5–8 ng/ml beyond one year. Mycophenolate mofetil is administered at an initial dose of 0.5–1.0 g every 12 h. Methylprednisolone is given intravenously during surgery at 10–15 mg/kg, followed by 250–500 mg/day intravenously for the first three days postoperatively. From the fourth day onwards, oral administration begins at 10–30 mg/day, gradually tapering to 10–15 mg/day by the 30th day post-surgery. During the maintenance phase, a low-dose regimen is employed, typically 10 mg/day at 2–3 months, 5–10 mg/day at 6 months, and 5.0–7.5 mg/day thereafter.

### Statistical analysis

Statistical analyses were performed using SPSS V25 (IBM Corp., Armonk, NY, USA) Continuous variables are determined by independent *t*-test or Mann–Whitney *U* test and expressed as average ± *SD* or median with quartile range, respectively. Classification variables are evaluated using chi-square test or Fisher’s exact test and expressed as numbers (percentages). All the statistical tests and confidence intervals are bilateral, *p* < 0.05 is considered to be statistically significant. This study utilized SPSS to calculate the survival rates of recipients and grafts in both diabetic and non-diabetic donor groups. Additionally, the survival rates of recipients and grafts corresponding to different RPS grades within the diabetic donor group were computed. Finally, Kaplan–Meier curves were plotted.

First of all, a univariate Logistic risk regression model was established for postoperative DGF in recipients using diabetic donor kidneys. Then, the variables with *p* < 0.1 were incorporated into the multi-factor Logistic risk regression model. Independent risk factors for postoperative DGF in recipients using diabetic donor kidneys were evaluated by risk models.

## Results

### Cohort characteristics

**Figure 1.**
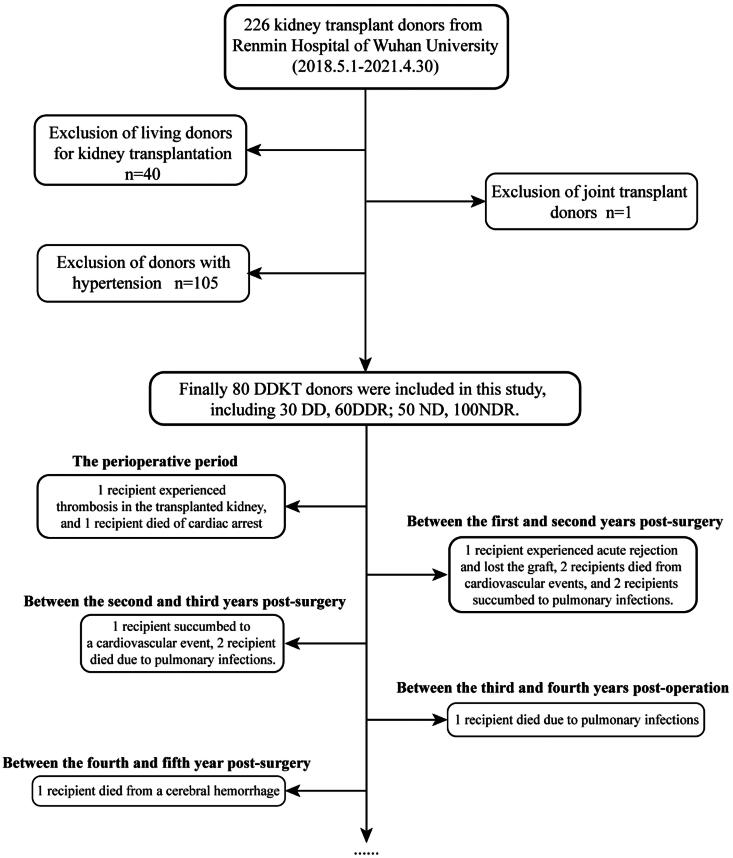
The subject selection process and recipient outcomes during follow-up in this study.

This study analyzed 226 kidney transplant donors from Renmin Hospital of Wuhan University between 1 May 2018 and 30 April 2021. We excluded 40 cases of living-donor kidney transplants (LDKT) and one case of heart-kidney combined transplant recipients. Additionally, 105 DDKT donors with preexisting hypertension were excluded. Consequently, 80 DDKT donors and 160 corresponding recipients were included in the study. These were divided into 30 diabetic donors (DD) and their recipients (DDR), and 50 control donors (ND) and their corresponding recipients (NDR). The flowchart for screening study subjects and the mortality of all recipients during the follow-up period are illustrated in Figure 1.

### Comparison of basic data of donors and recipients between diabetic renal donor group and control group

The analysis of the basic data of diabetic donors and control donors showed that there was no significant difference in age, BMI, and HBV infection between the two groups. The gender composition of the diabetic donor group was higher than that of the control group [29 (96.7%) *vs.* 42 (84.0%), *p* = 0.083), but there is no statistical difference.

Compared with the control group, the age, male proportion, BMI, transplant times, and HBV infection of diabetic donor recipients were similar. In addition, there was no significant difference in the number of human leucocyte antigen (HLA) mismatches (*p* = 0.052) and PRA > 10% (*p* = 0.963) between the diabetic donor group and the control group. Overall, there was no significant difference in basic data between the diabetic donor group and the control group (Table 1).

**Table 1.** Analysis of the baseline clinical information of the donor and recipient groups.

Variable	All	Diabetes donor	Normal donor	*p*‐Value
Donor	*n* = 80	*n* = 30	*n* = 50	
Age (year)	54.51 (±9.35)	53.57 (±8.89)	55.08 (±9.65)	0.487
Male (*n*)	71 (88.8%)	29 (96.7%)	42 (84.0%)	0.083
BMI (kg/m²)	22.92 (±4.25)	22.87 (±5.53)	22.94 (±3.31)	0.942
HBV (*n*)	14 (17.5%)	5 (16.7%)	9 (18.0%)	0.879
Recipient	*n* = 152	*n* = 58	*n* = 94	
Age (year)	43.38 (±10.88)	44.17 (±10.75)	42.91 (±10.98)	0.942
Male (*n*)	114 (71.3%)	44 (73.3%)	70 (70.0%)	0.654
BMI (kg/m²)	22.15 (±3.20)	21.95 (±2.82)	22.27 (±3.42)	0.544
HBV (*n*)	27 (16.9%)	13 (21.7%)	14 (14.0%)	0.212
HLA mismatches	3.96 (±0.98)	4.15 (±0.88)	3.84 (±1.02)	0.052
PRA > 10% (*n*)	43 (28.3%)	16 (27.6%)	27 (28.7%)	0.963
First kidney transplantation (*n*)	145 (95.4%)	54 (93.1%)	91 (96.8%)	0.290

BMI: body mass index; HBV: hepatitis B virus; HLA: human leucocyte antigen; PRA: panel reactive antibodies.

Age, BMI, and HLA mismatches were expressed as mean ± *SD*. Male, HBV, PRA > 10%, and first kidney transplantation were expressed as number (*n*) and percentage (%).

### Donor

**Figure 2.**
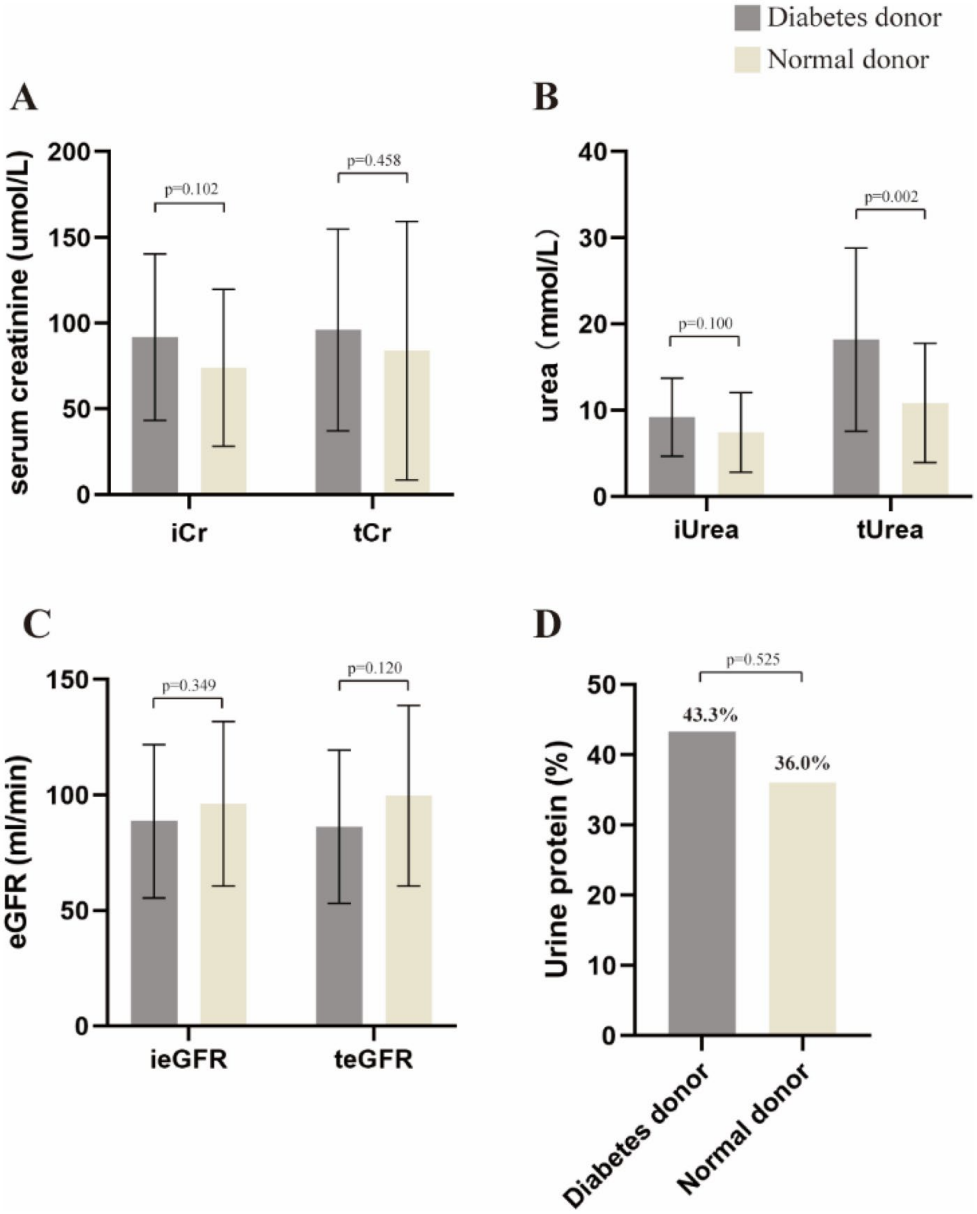
Biochemical information of diabetic and non-diabetic donors at the time of admission and prior to collection. (A) Initial serum creatinine at admission and terminal serum creatinine; (B) initial urea at admission and terminal urea; (C) initial eGFR at admission and terminal eGFR; (D) the proportion of urine protein positive people who obtained previous donors.

This study compiled donor information, including biochemical indicators measured from ICU admission until the final assessment before donation. At admission, diabetic donors exhibited elevated levels of Cr and Urea, along with a reduced eGFR, compared to the control group (Figures 2(A–C)). Although these differences did not achieve statistical significance, they suggest a potential impairment in renal function among diabetic donors relative to their non-diabetic counterparts. An analysis of terminal Cr, Urea, eGFR, and urine protein levels revealed a significant increase in terminal Urea in diabetic donors compared to the control group [18.20 ± 10.63 *vs.* 10.86 ± 6.92, *p* = 0.002]. No significant differences were observed between the groups concerning terminal albumin, hemoglobin, and urine protein ratios (Figures 2(D–F)).

**Figure 3.**
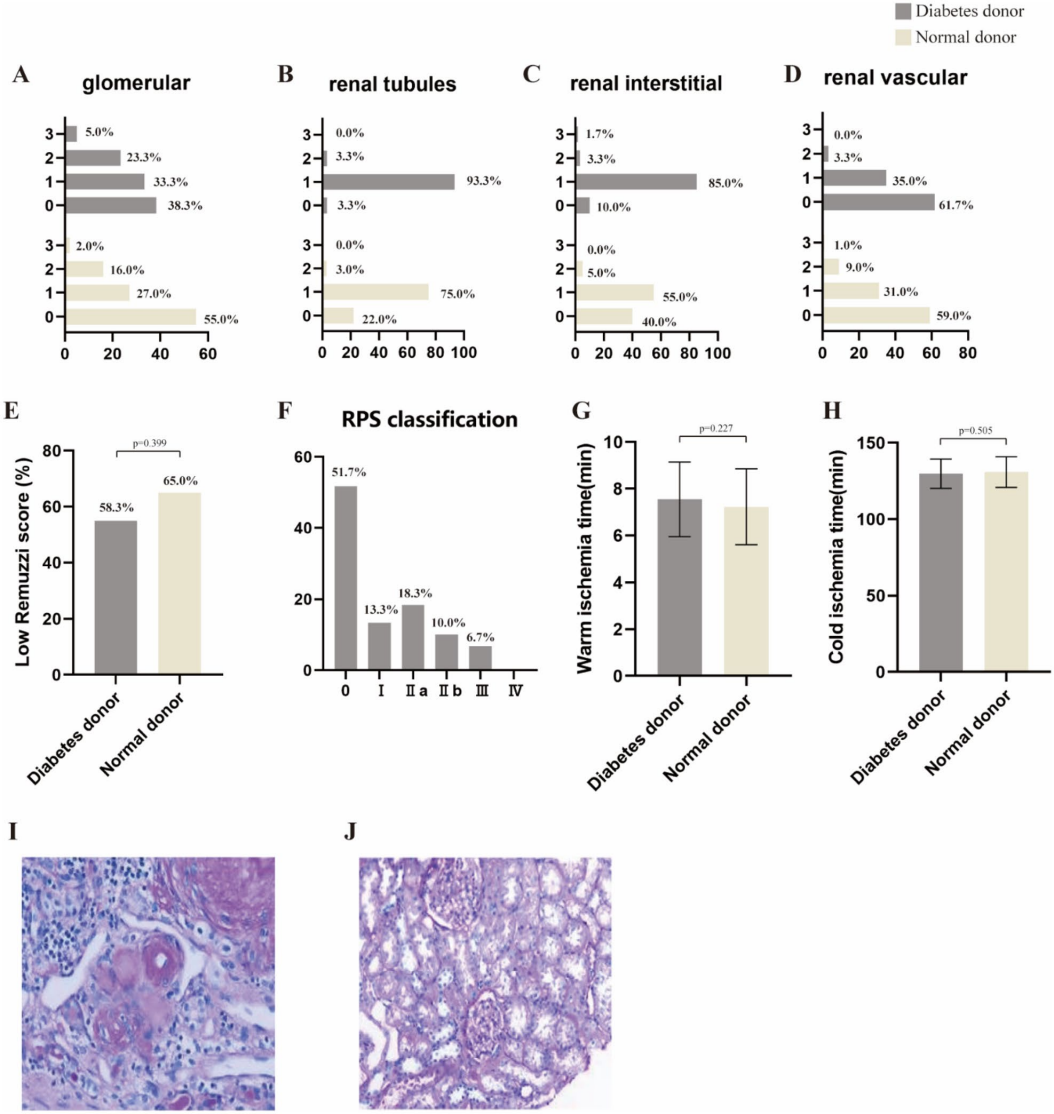
Renal pathological information of diabetic donors. (A) Glomerular Remuzzi pathological score of donor kidneys in two groups; (B) renal tubular Remuzzi pathological score of donor kidneys in two groups; (C) renal interstitial Remuzzi pathological score of donor kidneys in two groups; (D) renal vascular Remuzzi pathological score of donor kidneys in two groups; (E) proportion of kidney in donor low Remuzzi score group; (F) Renal Pathology Society rating of diabetic donor kidneys and the proportion of all levels of kidneys; (G) comparative analysis of warm ischemia time in kidney transplantation between the diabetic donor group and the normal donor group; (H) comparison analysis of cold ischemia time for kidney transplantation between the diabetic donor group and the normal donor group; (I) HE staining of donor kidney in control group; (J) HE staining of kidney from diabetic donors.

After the bilateral kidneys were obtained, the donor kidneys were scored by Remuzzi. The average level of glomerular score (*p* = 0.033), renal tubule score (*p* = 0.004), and renal interstitial score (*p* < 0.001) of donors in the control group was lower than that of diabetic donors (Figures 3(A–C)). However, there was no significant difference in renal vascular score between the two groups (Figure 3(D)). According to the score, the donated kidneys were divided into low score group (0–3) and high score group (>4). The results showed that there was no significant difference in kidney proportion between the low score group of diabetic donors and the control group (Figure 3(E)). At the same time, donor kidneys with scores >6 were only found in the diabetic donor group.

The kidneys from 60 diabetic donors were assessed using RPS grading. Of these, 51.7% exhibited no pathological changes, 13.3% were classified as grade I, 18.3% as grade IIa, 10% as grade IIb, 6.7% as grade III, and none as grade IV (Figure 3(F)). There were no significant differences in warm ischemia time or cold ischemia time between the diabetic donor group and the normal donor group in kidney transplantation (Figures 3(G,H)). Histological examination of kidney sections stained with HE revealed glomerulosclerosis in some diabetic donors (Figure 3(I)), whereas no pathological changes were observed in the control group (Figure 3(J)).

Detailed data information is shown in Table 2.

**Table 2.** Comparison of biochemical and renal pathological information between diabetic donors and control donors prior to acquisition.

	All	Diabetes donor	Normal donor	*p*‐Value
Donor	*n* = 80	*n* = 30	*n* = 50	
iCr (umol/L)	80.75 ± 47.31	91.93 ± 48.51	74.04 ± 45.76	0.102
iUrea (mmol/L)	8.10 ± 4.62	9.20 ± 4.51	7.44 ± 4.61	0.100
ieGFR (ml/min)	93.36 ± 34.72	88.63 ± 33.13	96.19 ± 35.66	0.349
iALB (g/L)	35.49 ± 6.73	36.96 ± 8.37	34.60 ± 5.42	0.130
iHB (g/L)	115.59 ± 28.82	120.43 ± 31.18	112.68 ± 27.21	0.246
tCr (umol/L)	88.53 ± 69.52	96.03 ± 58.82	84.02 ± 75.43	0.458
tUrea (mmol/L)	13.61 ± 9.16	18.20 ± 10.63	10.86 ± 6.92	0.002
teGFR (ml/min)	94.68 ± 37.29	86.30 ± 33.15	99.70 ± 39.13	0.120
tALB (g/L)	35.76 ± 4.61	34.71 ± 4.19	36.39 ± 4.78	0.116
tHB (g/L)	106.95 ± 23.82	112.83 ± 27.53	103.42 ± 20.79	0.112
Urine protein (*n*)	31 (38.8%)	13 (43.3%)	18 (36.0%)	0.525
Remuzzi score	*n* = 160	*n* = 60	*n* = 100	
Glomerular (*n*)				0.033
0	78 (48.8%)	23 (38.3%)	55 (55.0%)	
1	47 (29.4%)	20 (33.3%)	27 (27.0%)	
2	30 (18.8%)	14 (23.3%)	16 (16.0%)	
3	5 (3.1%)	3 (5.0%)	2 (2.0%)	
Renal tubules (*n*)				0.004
0	24 (15.0%)	2 (3.3%)	22 (22.0%)	
1	131 (81.9%)	56 (93.3%)	75 (75.0%)	
2	5 (3.1%)	2 (3.3%)	3 (3.0%)	
3	0 (0.0%)	0 (0.0%)	0 (0.0%)	
Renal interstitial (*n*)				<0.001
0	46 (28.8%)	6 (10.0%)	40 (40.0%)	
1	106 (66.3%)	51 (85.0%)	55 (55.0%)	
2	7 (4.4%)	2 (3.3%)	5 (5.0%)	
3	1 (0.6%)	1 (1.7%)	0 (0.0%)	
Renal vascular (*n*)				0.528
0	96 (60.0%)	37 (61.7%)	59 (59.0%)	
1	52 (32.5%)	21 (35.0%)	31 (31.0%)	
2	11 (6.9%)	2 (3.3%)	9 (9.0%)	
3	1 (0.6%)	0 (0.0%)	1 (1.0%)	
Low Remuzzi score (*n*)	98 (61.3%)	33 (55.0%)	65 (65.0%)	0.211
Remuzzi score >6 (*n*)	2 (1.3%)	2 (3.3%)	0 (0.0%)	0.159
RPS grading		*n* = 60		
0		31 (51.7%)		
I		8 (13.3%)		
IIa		11 (18.3%)		
IIb		6 (10.0%)		
III		4 (6.7%)		
IV		0 (0.0%)		
Warm ischemia time (min)	7.35 ± 1.618	7.55 ± 1.599	7.23 ± 1.626	0.227
Cold ischemia time (min)	130.47 ± 9.878	129.80 ± 9.669	130.88 ± 10.029	0.505

iCr: initial serum creatinine at admission; iUrea: initial urea at admission; ieGFR: initial estimated glomerular filtration rate at admission; iALB: initial albumin at admission; iHB: initial hemoglobin at admission; tCr: terminal serum creatinine; tUrea: terminal urea; teGFR: terminal estimated glomerular filtration rate; tALB: terminal albumin; tHB: terminal hemoglobin; Urine protein: terminal urine protein; Low Remuzzi score: according to the Remuzzi score of both kidneys of the donor, 0–3 was a low score. RPS grading: Renal Pathology Society grading.

iCr, iUrea, ieGFR, iALB, iHB, tCr, tUrea, teGFR, tALB, tHB, warm ischemia time, and cold ischemia time were expressed as mean ± *SD*. Number (*n*) and percentage (%) of men expressed as urine protein, Remuzzi score, low Remuzzi score, Remuzzi score >6, and RPS grading.

### Recipient

**Figure 4.**
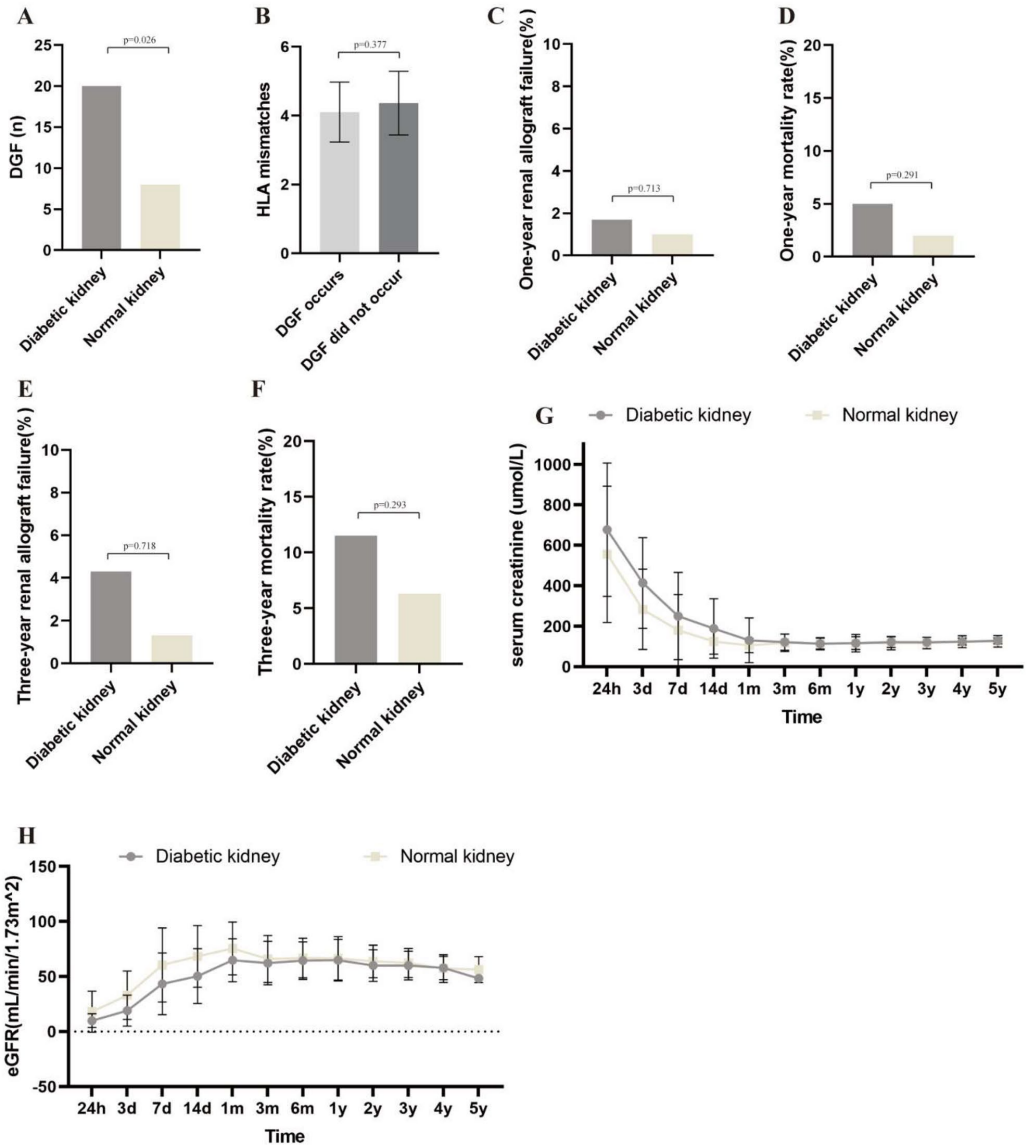
Postoperative information of recipients using diabetic and non-diabetic donor kidneys. (A) The proportion of recipients with DGF in the two groups; (B) a comparative analysis of HLA mismatch numbers between recipients with DGF and those without DGF in the diabetic donor group; (C) 1 year post-operation, the graft failure rates in the two recipient groups; (D) the mortality rates of recipients in both groups 1 year post-surgery; (E) 3 years post-operation, the graft failure rates in the two recipient groups; (F) the mortality rates of recipients in both groups 3 years post-surgery; (G) the changes of serum creatinine in both groups at 24 h, 3 days, 7 days, 14 days, 1 month, 3 months, 6 months, 1 year, 2 years, 3 years, 4 years, and 5 years; (H) the changes of eGFR in both groups at 24 h, 3 days, 7 days, 14 days, 1 month, 3 months, 6 months, 1 year, 2 years, 3 years, 4 years, and 5 years.

As of 30 April 2023, 35 recipients were followed up to the fifth year, 37 recipients were followed up to the fourth year, 47 recipients were followed up to the third year, and 28 recipients were followed up to the second year. The average follow-up period was 42.145 ± 1.071 months. There were no significant differences in postoperative hemoglobin and transaminase levels between the DDR and NDR groups. However, the incidence of DGF postoperatively was significantly higher in the DDR group compared to the NDR group [20 *vs.* 8%, *p* = 0.026] (Figure 4(A)).

Statistical analysis of HLA mismatch numbers between recipients with and without DGF in the DDR group indicated that the HLA mismatch count in recipients using kidneys from diabetic donors did not affect the occurrence of postoperative DGF (Figure 4(B)). The graft failure rate at 1 year post-surgery [1.7% vs 1.0%, p=0.713] (Figure 4(C)) and mortality rate at 1 year [5.0% vs 2.0%, p=0.291] (Figure 4(D)) showed no significant differences between the two groups of recipients. Similarly, no significant differences were observed in the graft failure rate at 3 years [1.7% vs 1.0%, p=0.713] (Figure 4(E)) or the mortality rate at 3 years [5.0% vs 2.0%, p=0.291] (Figure 4(F)). These findings indicate that both recipient groups have comparable short-term and long-term prognoses.

Notably, although the blood creatinine levels decreased in both groups postoperatively, the DDR group had significantly higher levels at 24 h [677.37 ± 329.47 *vs.* 556.02 ± 336.20, *p* = 0.027], 3 days [414.02 ± 223.60 *vs.* 283.47 ± 197.98, *p* < 0.001], 7 days [250.33 ± 214.82 *vs.* 181.47 ± 175.95, *p* = 0.029], 14 days [189.60 ± 147.07 *vs.* 125.06 ± 61.90, *p* < 0.001], and 1 month [131.00 ± 110.76 *vs.* 105.42 ± 35.12, *p* = 0.035] compared to the NDR group. However, this difference gradually diminished over time, and by the third postoperative month [122.12 ± 39.69 *vs.* 118.97 ± 43.19, *p* = 0.649], there was no significant difference in serum creatinine levels between the two groups. The eGFR analysis revealed a similar trend. Although the recovery of renal function after DDR was slower compared to NDR, both groups achieved the same level of renal function recovery by the third month (Figures 4(G,H)).

The three-year mortality rates [11.5 *vs.* 6.3%, *p* = 0.293] and three-year graft failure rates [4.3 *vs.* 1.3%, *p* = 0.718] between the two recipient groups showed no significant differences, indicating that the long-term prognosis for DDR is comparable to that of NDR.

Detailed data information is shown in Table 3.

**Table 3.** Comparison of renal function changes and short-term and long-term outcomes between the two groups of recipients post-surgery.

Variable	All	Diabetic kidney	Normal donor	*p*‐Value
Recipient	*n* = 160	*n* = 60	*n* = 100	
HB (g/L)	101.9 ± 18.26	101.12 ± 14.16	102.37 ± 20.38	0.676
ALT (U/L)	11.44 ± 4.87	11.47 ± 5.33	11.43 ± 4.59	0.963
AST (U/L)	15.21 ± 5.86	14.78 ± 5.32	15.47 ± 6.17	0.474
24hCr (umol/L)	601.52 ± 337.93	677.37 ± 329.47	556.02 ± 336.20	0.027
24heGFR (mL/min/1.73m^2)	15.03 ± 15.69	9.94 ± 6.24	18.09 ± 18.63	0.001
3dCr (umol/L)	332.43 ± 216.74	414.02 ± 223.60	283.47 ± 197.98	<0.001
3deGFR (mL/min/1.73m^2)	27.80 ± 20.47	19.12 ± 13.97	33.00 ± 22.00	<0.001
7dCr (umol/L)	207.29 ± 193.70	250.33 ± 214.82	181.47 ± 175.95	0.029
7deGFR (mL/min/1.73m^2)	54.21 ± 32.69	43.47 ± 28.10	60.65 ± 33.68	0.001
	*n* = 158	*n* = 59	*n* = 99	
14dCr (umol/L)	149.41 ± 106.95	189.60 ± 147.07	125.06 ± 61.90	<0.001
14deGFR (mL/min/1.73m^2)	61.74 ± 28.16	50.49 ± 24.85	68.44 ± 27.98	<0.001
1mCr (umol/L)	115.08 ± 74.17	131.00 ± 110.76	105.42 ± 35.12	0.035
1meGFR (mL/min/1.73m^2)	71.58 ± 22.95	64.89 ± 19.55	75.56 ± 23.97	0.004
1mUrea (mmol/L)	11.20 ± 6.58	11.78 ± 5.95	10.85 ± 6.94	0.389
3mCr (umol/L)	120.15 ± 41.81	122.12 ± 39.69	118.97 ± 43.19	0.649
3meGFR (mL/min/1.73m^2)	64.67 ± 20.78	62.28 ± 19.77	66.10 ± 21.33	0.265
	*n* = 157	*n* = 59	*n* = 98	
6mCr (umol/L)	113.98 ± 29.00	114.37 ± 27.00	113.74 ± 30.31	0.895
6meGFR (mL/min/1.73m^2)	66.07 ± 17.56	64.57 ± 16.93	66.98 ± 17.95	0.407
	*n* = 153	*n* = 56	*n* = 97	
1yCr (umol/L)	116.98 ± 38.84	117.12 ± 31.62	116.89 ± 42.85	0.972
1yeGFR (mL/min/1.73m^2)	65.99 ± 19.37	64.92 ± 18.89	66.60 ± 19.72	0.606
DGF (*n*)	20 (12.5%)	12 (20.0%)	8 (8.0%)	0.026
One-year renal allograft failure (*n*)	2 (1.3%)	1 (1.7%)	1 (1.0%)	0.713
One-year mortality rate (*n*)	5 (3.3%)	3 (5.0%)	2 (2.0%)	0.291
Three-year renal allograft failure (*n*)	2 (1.6%)	1 (4.3%)	1 (1.3%)	0.718
Three-year mortality rate (*n*)	11 (9.0%)	6 (11.5%)	5 (6.3%)	0.293

HB: hemoglobin 24 h after operation; ALT: alanine aminotransferase 24 h after operation; AST: aspartate aminotransferase 24 h after operation; 24hCr: serum creatinine 24 h after operation; 24heGFR: estimated glomerular filtration rate 24 h after operation; 3dCr: serum creatinine 3 days after operation; 3deGFR: estimated glomerular filtration rate 3 days after operation; 7dCr: serum creatinine 7 days after operation; 7deGFR: estimated glomerular filtration rate 7 days after operation; 14dCr: serum creatinine 14 days after operation; 14deGFR: estimated glomerular filtration rate 14 days after operation; 1mCr: serum creatinine 1 month after operation; 1meGFR: estimated glomerular filtration rate 1 month after operation; 3mCr: serum creatinine 3 months after operation; 3meGFR: estimated glomerular filtration rate 3 months after operation; 6mCr: serum creatinine 6 months after operation; 6meGFR: estimated glomerular filtration rate 6 months after operation; 1yCr: serum creatinine 1 year after operation; 1yeGFR: estimated glomerular filtration rate 1 year after operation; DGF: delayed graft function.

HB, ALT, AST, 24hCr, 3dCr, 7dCr, 14dCr, 1mCr, 1mUrea, 3mCr, 6mCr, and 12mCr were expressed as mean ± *SD*. Number (*n*) and percentage (%) of men expressed as DGF, renal allograft failure, and mortality rate within 3 year.

### The impact of kidneys from diabetic donors with different RPS grades on recipients

**Figure 5.**
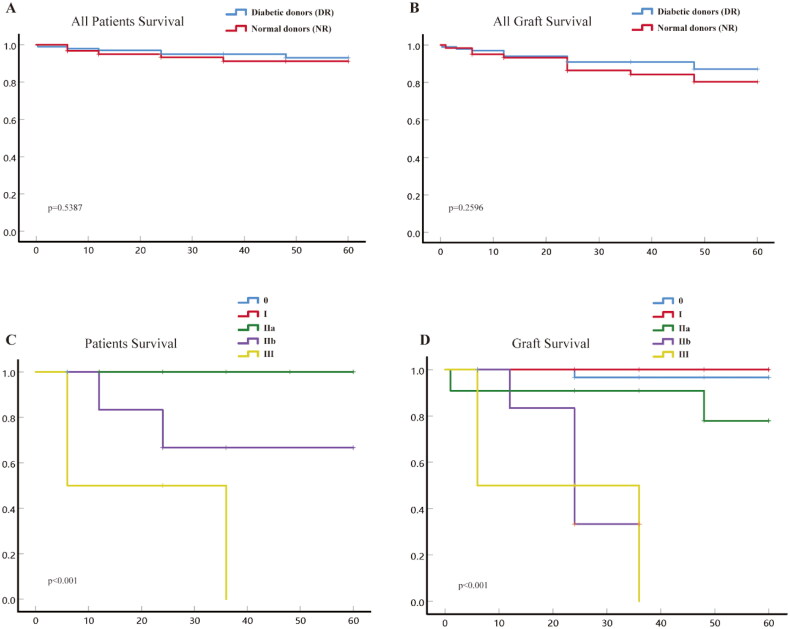
The Kaplan–Meier curve analysis of recipients and grafts from both groups, as well as recipients and grafts corresponding to different RPS grades within the diabetic donor group, was conducted. (A) The Kaplan–Meier curve of recipients in the diabetic donor group and the normal donor group are illustrated below; (B) the Kaplan–Meier curve of grafts from the diabetic donor group and the normal donor group; (C) Kaplan–Meier curve analysis of recipients corresponding to different RPS grades within the diabetic donor group; (D) the Kaplan–Meier curve analysis of grafts corresponding to different RPS grades within the diabetic donor group.

Kaplan–Meier curve analysis was performed to compare the survival rates of recipients and grafts between two groups. The results indicated that the use of diabetic donor kidneys had no significant impact on the survival rates of recipients and grafts (Figures 5(A,B)). However, stratified analysis based on the RPS grading of diabetic donor kidneys revealed that transplantation with grade IIb and III kidneys significantly increased recipient mortality and graft failure rates (Figures 5(C,D)).

### Analysis of risk factors of postoperative DGF in diabetic renal donors

First, single-factor logistic regression was used to analyze the impact of each independent variable on the occurrence of DGF in recipients of diabetic kidney donors. The variables with *p* < 0.1 included donor terminal Cr, donor terminal eGFR, recipient gender, and recipient PRA. The selected variables were put into multi-factor Logistic regression analysis model. Finally, it is concluded that recipient gender [OR: 5.452 (1.330–22.353), *p* = 0.013] and pre-transplant PRA positivity [OR: 34.879 (7.698–158.030), *p* < 0.001] are independent risk factors for postoperative DGF. Table 4 provides detailed analysis results.

**Table 4.** Logistic regression analysis of risk factors for delayed graft function in recipients of kidneys from diabetic donors.

Variable	Single factor logistic regression OR (95%CI)	*p*‐Value	Multivariate logistic regression OR (95%CI)	*p*‐Value
Donor tCr	1.008 (1.001–1.015)	0.031		
Donor teGFR	0.984 (0.969–0.998)	0.029		
Recipient gender	2.879 (1.098–7.549)	0.032	5.452 (1.330–22.353)	0.013
HLA mismatch	1.432 (0.820–2.501)	0.207		
Recipient PRA > 10%	13.667 (4.639–40.260)	<0.001	34.879 (7.698–158.030)	<0.001

OR: odds ratio; 95% CI: 95% confidence interval; tCr: terminal serum creatinine; teGFR: terminal estimated glomerular filtration rate; HLA: human leucocyte antigen; PRA: panel reactive antibodies.

## Discussion

**Figure 6.**
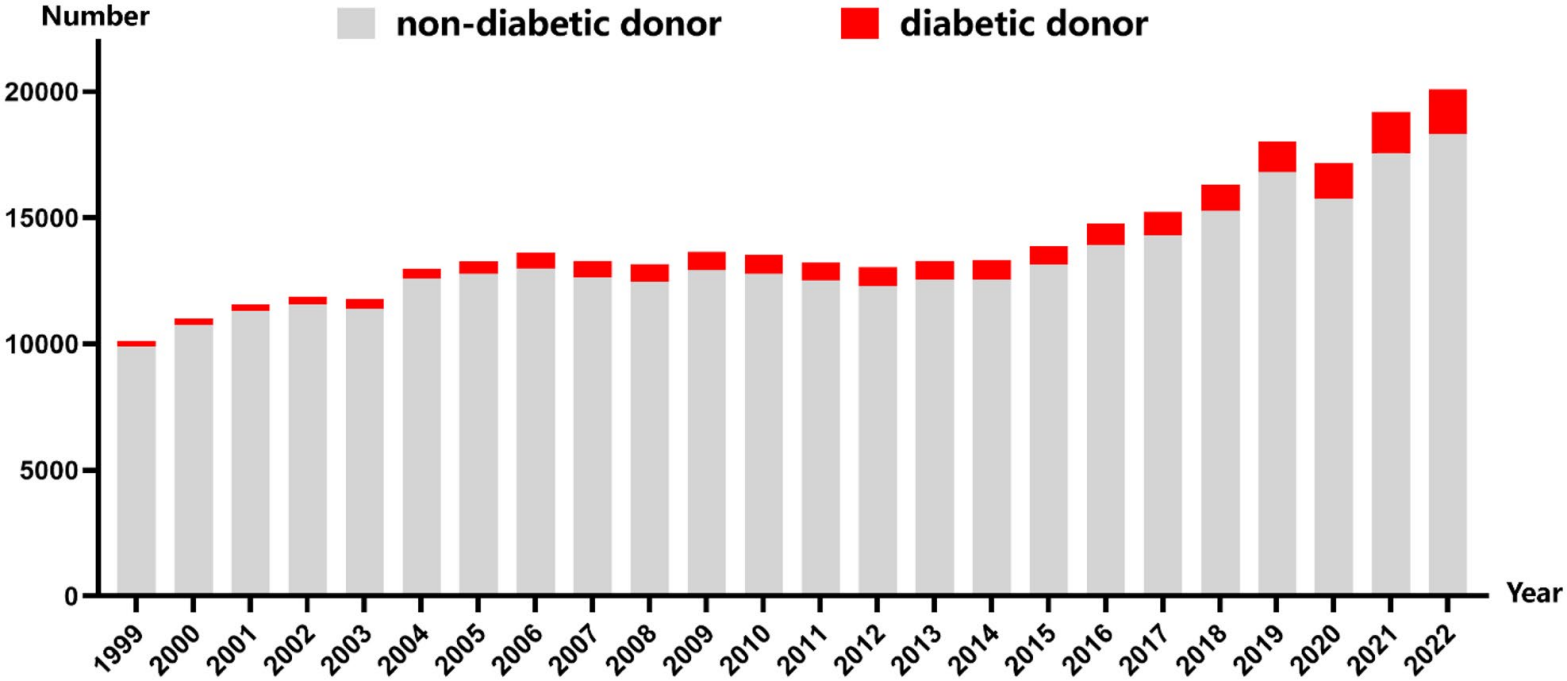
Proportion of diabetic donors for kidney transplantation in the United States from 1999 to 2022.

Traditionally, standard donors have been considered the ideal candidates for kidney transplantation, while a history of diabetes has been regarded as a relative contraindication for kidney donors. However, as the number of end-stage renal disease (ESRD) patients continues to rise annually, the availability of suitable kidney donors is increasingly limited. Consequently, transplant centers are working to broaden the criteria for standard donors. Additionally, the incidence of diabetes is on the rise, affecting even younger populations. Analysis of the United Network for Organ Sharing (UNOS) database reveals a gradual increase in the proportion of diabetic donors in kidney transplantation (Figure 6). This trend compels us to evaluate whether kidneys from diabetic donors significantly impact both short-term and long-term allograft function and recipient survival. A study utilizing UNOS data revealed that, despite the graft survival rate being lower for diabetic donors compared to non-diabetic standard donors, it was still markedly superior to that of non-diabetic extended standard donors [24]. Furthermore, several studies have indicated that there is no significant difference in the rates of postoperative rejection, long-term graft survival, or patient survival between kidneys from diabetic donors and those from non-diabetic donors [25,26]. Currently, there is no definitive answer regarding whether the recipient’s blood glucose status impacts the survival rate of diabetic donor kidneys. Research by Khan et al. indicates that diabetic recipients face a 2- to 3-fold increased risk of mortality compared to their non-diabetic counterparts [27]. However, Gilbert et al. argue that the renal survival rate of diabetic donors is not influenced by the diabetic status of the recipients [28]. There is no correlation between the duration of donor diabetes and the severity of donor diabetic nephropathy. Additionally, it does not serve as an independent risk factor for graft survival post-operatively [27,29]. Previous studies have demonstrated that 10 years after pancreas transplantation, nephropathy in diabetic patients was reversed, with the structure of glomeruli, renal tubules, and renal interstitium largely restored to normal [30,31]. This suggests that mitigating the preexisting hyperglycemic conditions could slow the progression of subtle pathological changes in the kidneys of diabetic donors. Consequently, this study ensured meticulous control of postoperative blood glucose levels for all recipients.

Numerous studies have investigated the influence of diabetic donor kidneys on long-term graft survival and recipient outcomes. This study retrospectively examined data from diabetic donor kidney transplants (DDKT) and their corresponding recipients at Wuhan University People’s Hospital between 1 May 2018 and 30 April 2021. The aim was to evaluate the effects of diabetic donor kidneys on postoperative complications, renal function, and both short-term and long-term outcomes. There were no significant differences in basic characteristics, such as age, gender, and BMI between diabetic and non-diabetic kidney donors. However, terminal urea levels in diabetic donor kidneys were significantly elevated compared to those in the control group (*p* = 0.002). Although there was no statistically significant difference in the overall Remuzzi pathological scores between the two donor groups, diabetic donor kidneys displayed more pronounced glomerulosclerosis, tubular atrophy, and interstitial fibrosis compared to their non-diabetic counterparts. Additionally, kidneys with Remuzzi scores >6 were found exclusively in the diabetic donor group. RPS grading of kidneys from diabetic donors showed that 65% were classified as grade 0 or I, 6.7% as grade III, and none as grade IV.

DGF is one of the most prevalent early complications following renal transplantation. It is linked to readmission within 30 days post-transplant, acute rejection, and transplant failure [32]. The analysis of this study revealed that the incidence of delayed graft function (DGF) was significantly higher in the diabetic renal donor group compared to the control group (*p* = 0.026). Both univariate and multivariate logistic regression analyses were employed to identify factors influencing postoperative DGF in recipients from the diabetic donor group. The results indicated that recipient gender and a pre-transplant PRA > 10% were independent predictors of DGF. Previous research has demonstrated that male recipients are more likely to experience postoperative DGF compared to female recipients [33]. However, our analysis revealed that recipients of kidneys from diabetic donors exhibited a 5.452-fold increased likelihood of developing delayed graft function (DGF) if they were women compared to men. This finding may be attributed to the limited sample size within the diabetic donor group. To validate these results, it is essential to involve additional transplant centers and expand the sample size. Additionally, a panel reactive antibody (PRA) level >10% triples the recipient’s risk of transplant complications [34]. At the same time, elevated PRA levels are associated with increased rejection rates in renal transplant recipients [35]. We observed that a pre-transplant PRA level >10% is linked to an increased risk of delayed graft function (DGF) in recipients of kidneys from diabetic donors. This indicates that for recipients receiving kidneys from diabetic donors, it is crucial to more rigorously manage PRA levels before the transplant procedure.

Analyzing the postoperative data from the two groups of recipients yielded an intriguing finding. Following renal transplantation, serum creatinine levels in the recipients decreased over time. Data indicated that at various time points within the three months post-operation, the serum creatinine levels in the diabetic renal donor group were elevated compared to the control group. However, the disparity between the two groups gradually diminished over time. By the third month post-operation, no significant difference in serum creatinine levels was observed between the groups. This implies that while donor diabetes can impact the functional recovery of the transplanted kidney, its effects are confined to the initial three months after surgery. Notably, kidney disease in diabetic patients was reversed ten years after pancreas transplantation. In contrast, early diabetic nephropathy changes in the diabetic donor kidney showed improvement one year after recipient transplantation [36]. Regardless of whether the recipient had diabetes or stringent blood sugar control, the kidney disease from the diabetic donor did not exacerbate [37]. The serum creatinine levels in the recipient indicated that, after the diabetic donor’s kidney was placed in a normoglycemic environment, its renal function normalized to levels comparable to those of kidneys from non-diabetic donors within three months. Conversely, pathological changes in the kidney typically require at least one year to manifest.

In this study, RPS grading was employed to evaluate the postoperative survival of recipients with kidneys from diabetic donors. Our findings revealed a significant reduction in postoperative survival rates for recipients receiving kidneys graded III, whereas no significant differences were observed among grades 0, I, and IIa. These results corroborate those of Truong et al. indicating that kidneys with an RPS grade of ≥ III from diabetic donors are not recommended [38]. This significantly increases the recipient’s risk of postoperative death.

There was no significant difference in the incidence of renal allograft failure or mortality between the two groups within three years post-operation. This suggests that the use of kidneys from diabetic donors does not adversely affect graft survival or patient longevity. For end-stage renal disease (ESRD) patients who have been on the waiting list for an extended period, receiving kidneys from deceased diabetic donors can significantly lower the risk of mortality and reduce both the economic burden on patients and the societal costs [39]. Based on the findings of this study, it is recommended that transplant centers consider accepting kidneys from evaluated diabetic donors to a greater extent, as this could potentially save more lives.

Furthermore, this study has several limitations. While it was not affected by selection bias, it was constrained by stringent inclusion criteria, resulting in only one female diabetic donor. This limitation may influence the evaluation of gender-related factors in diabetic donors on recipient outcomes. Additionally, Melk et al. found that donor age modifies the relationship between donor gender and graft survival rate [40]. In terms of donor gender alone, there seems to be no substantial evidence indicating an early impact on recipients [41]. We will continue to collect kidney transplant data from this center and conduct further research on the influence of donor gender on recipient and graft survival.

## Conclusion

Overall, there was no significant difference in the overall mass of the kidneys between diabetic and non-diabetic donors, but the former kidneys had more severe glomerulosclerosis, tubular atrophy, and interstitial fibrosis. Recipients in the post-transplant diabetic donor group were more likely to develop DGF and had a slower decrease in serum creatinine after transplantation. However, there was no difference in renal function between the two groups 3 months after operation. The use of diabetic donor kidneys does not have a negative effect on long-term graft survival and survival. The study suggests that transplant centers can try to use more kidneys from assessed diabetes donors and monitor and care for recipients early after surgery to minimize the risk of using kidneys from diabetic donors.

## Data Availability

The original research data of this article involves patient privacy and therefore cannot be made public.
